# Traditional Chinese Medicine: Role in Reducing β-Amyloid, Apoptosis, Autophagy, Neuroinflammation, Oxidative Stress, and Mitochondrial Dysfunction of Alzheimer’s Disease

**DOI:** 10.3389/fphar.2020.00497

**Published:** 2020-04-22

**Authors:** Shi-Yu Chen, Yue Gao, Jia-Yi Sun, Xian-Li Meng, Dong Yang, Lin-Hong Fan, Li Xiang, Ping Wang

**Affiliations:** ^1^College of Pharmacy, Chengdu University of Traditional Chinese Medicine, Chengdu, China; ^2^Innovative Institute of Chinese Medicine and Pharmacy, Chengdu University of Traditional Chinese Medicine, Chengdu, China

**Keywords:** Alzheimer’s disease, traditional Chinese medicine, β-amyloid, apoptosis, autophagy, neuroinflammation, oxidative stress, mitochondrial dysfunction

## Abstract

Alzheimer’s disease (AD) is a progressive age-related neurodegenerative disease characterized by memory loss and cognitive impairment. The major characteristics of AD are amyloid β plaques, apoptosis, autophagy dysfunction, neuroinflammation, oxidative stress, and mitochondrial dysfunction. These are mostly used as the significant indicators for selecting the effects of potential drugs. It is imperative to explain AD pathogenesis and realize productive treatments. Although the currently used chemical drugs for clinical applications of AD are effective in managing the symptoms, they are inadequate to achieve anticipated preventive or therapeutic outcomes. There are new strategies for treating AD. Traditional Chinese Medicine (TCM) has accumulated thousands of years of experience in treating dementia. Nowadays, numerous modern pharmacological studies have verified the efficacy of many bioactive ingredients isolated from TCM for AD treatment. In this review, representative TCM for the treatment of AD are discussed, and among these herbal medicines, the Lamiaceae family accounts for the highest proportion. It is concluded that monomers and extracts from TCM have potential therapeutic effect for AD treatment.

## Introduction

**Figure 1:**
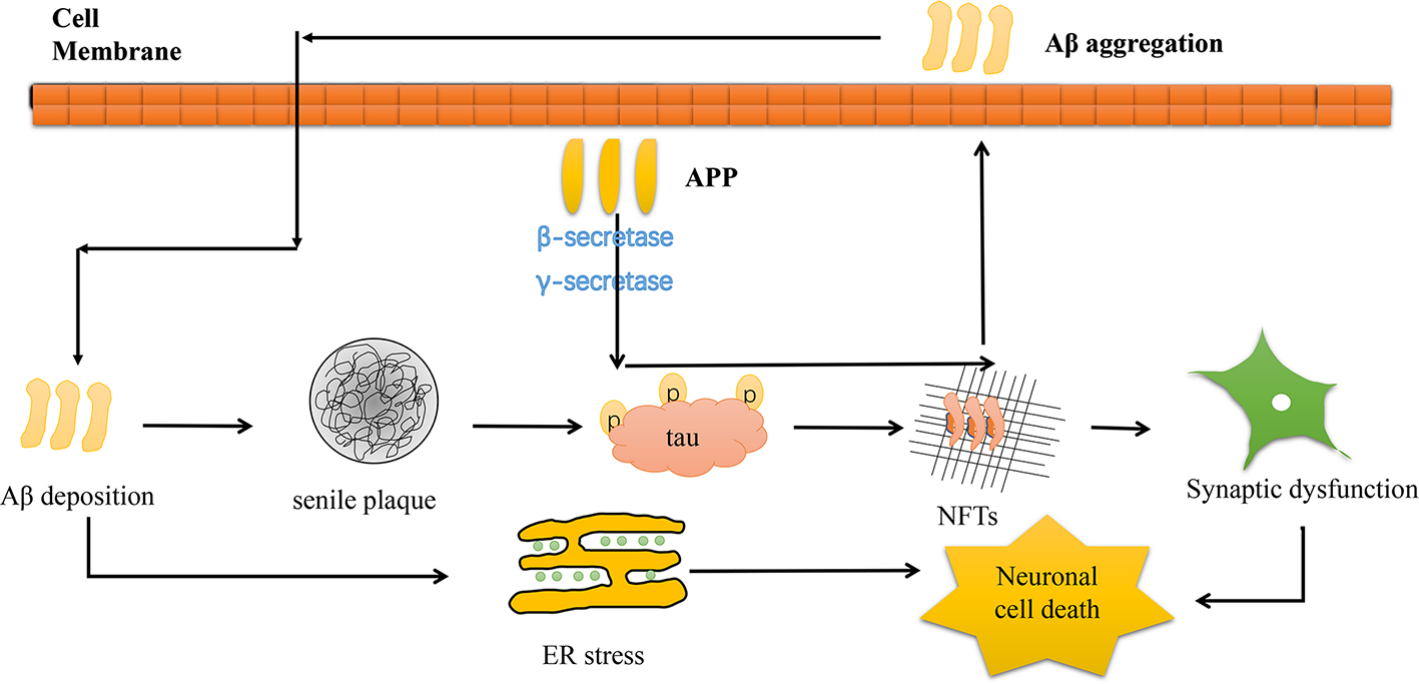
Schematic diagram depicting the pathogenesis of AD. Aβ generated from its precursor APP processing *via* cleavage by β-secretase and γ-secretase. Aβ deposition would cause senile plaque and further tau phosphorylation and aggregation to form NFTs, which lead to the loss of neurons and synaptic dysfunction. In addition, Aβ deposition induce mitochondrial injury and trigger ER stress, causing neuronal cell death.

Alzheimer’s disease (AD) is the most common neurodegenerative disease with high mortality in adults (Van Cauwenberghe et al., 2016; El Kadmiri et al., 2018). The prevalence of AD has been increasing, and age is considered the major risk factor (Alzheimer’s Association, 2018). Current studies show that, globally, 44 million people live with dementia. Moreover, that number is expected to triple by 2050 as the population ages and the AD onset tends to be younger ages (Lane et al., 2018). The symptoms of AD include cognitive impairments, memory loss, and executive function loss, hence a hefty burden to the society (Alzheimer’s Association, 2018). The pathogenesis of AD is characterized by β-amyloid plaques deposition, tau protein hyperphosphorylation, neuroinflammation, mitochondrial dysfunction, autophagy dysfunction, and oxidative stress (Choi, 1995). The main cause of AD is mutation in either of these three genes―amyloid precursor protein (APP), presenilin 1 (PSEN1) or presenilin 2 (PSEN2) gene. The symptoms of fAD generally occur earlier than sporadic AD between the age of 30 and 50 years (Bateman et al., 2011). A key component of extracellular senile plaque is amyloid β peptide (Aβ). Amyloid plaques are Aβ with 40 or 42 amino acids (Aβ40 and Aβ42) that cluster abnormally extracellularly. The two metabolites are produced by APP after β-secretase and γ-secretase cleavage (Fleisher et al., 2008; Fan et al., 2017). Once APP levels are abnormal, Aβ accumulates causing tau phosphorylation and aggregation to form neurofibrillary tangles (NFTs). NFTs are insoluble twisted fibers made up of clustered hyperphosphorylated tau proteins in AD neurons. Normally, tau pathology begins at the medial temporal lobe allocortex and then spreads to the united neocortex (Lindwall and Cole, 1984; Serrano-Pozo et al., 2011). The Aβ and tau pathology build up causes endoplasmic reticulum stress (ER stress), leading to synaptic dysfunction and AD neurodegeneration (Richardson et al., 2015) (**Figure 1**). Besides, a recent study revealed that Aβ and reduced glutamate reuptake levels can trigger hyperexcitation in sensitive neurons. Inactive neurons are resistant to Aβ-mediated hyperactivation, whereas hyperactivity occurs in active neurons. This hyperactivation vicious cycle can be maintained by Aβ-induced AD brain extracts and Aβ-dimers (Zott et al., 2019).

The current approved drugs for the clinical treatment of AD are cholinesterase inhibitors (ChEIs) and N-methyl-d-aspartic acid (NMDA) receptor antagonists (Sun et al., 2012). Donepezil, rivastigmine, galantamine, and memantine are usually used to treat AD, but these drugs are all single-target drugs. However, they display modest and transitory symptoms improvement accompanied by side effects and hardly prevents or reverses the disease (Silva et al., 2014). Therefore, it is necessary to find better drugs for AD treatment.

Traditional Chinese medicine (TCM) has been established in China health care system over thousands of years (Tang et al., 2008; Xu and Yang, 2009). It has played a very important role in the treatment of chronic diseases such as lung cancer, coronary heart diseases, allergy, diabetes, and infections (Li and Brown, 2009; Li et al., 2009; Gu et al., 2010; Shi et al., 2012; Guo et al., 2012; Liu et al., 2014; Jiang et al., 2016; Zhu et al., 2017). TCM is usually viewed as more accessible, affordable, and acceptable form of treatment, and nearly a quarter of all modern drugs are derived from natural products. Thus, TCM is the basis of primary health care system and innovative medicines (Chan et al., 2014). TCM has been frequently applied in the treatment of dementia and has shown exceptional advantages due to its multi-target, multi-system, multi-link, and multi-pathway capacity. There are numerous prescriptions for treating dementia in the historical records (Luo et al., 2014), such as; *Kaixin Powder, Naoling Decoction, Puzzle decoction, Huannaoyicong Decoction*, and *Compound Formula Rehmannia* that have a significantly improve intelligence, anti-fatigue, enhance immunity, delay senility, improve memory, prevent, and treat dementia without noticeable side effects (Zang et al., 2016). Recently, some scholars conducted a general analysis on twenty eligible studies with 1,767 subjects in eight database searches. These studies investigated the combined use or compared application of TCM and clinical drugs, such as donepezil. They found out that TCM as adjuvant therapy exhibited an additive anti-AD advantage and was mainly safe and well tolerated in AD patients. These properties are not in the present approved drugs (Yang et al., 2017). According to reported evidences focus on treating AD is more on early detection of the pre-symptomatic phase and the prevalence of early dementia signs. However, TCM has wealthy clinical impact and experience in the prevention and management of chronic diseases including AD. Therefore, there is need to focus on the therapeutic potential of TCM for AD treatment (Hügel, 2015). Active compounds extracted from TCM have therapeutic effects on AD *in vivo* and *in vitro*, and some TCM drugs have been applied in clinical trials, providing an approach for AD drug development. Modern pharmacological researches confirmed that bioactive compounds extracted from TCM such as morroniside, curcumin, triptolide, and berberine (Ber) have anti-AD activity (Gu et al., 2004; Fan et al., 2017; Huang et al., 2017; Chen et al., 2018b). Thus, TCM is a potential source of AD drug. Therefore, it is important to analyze and summarize the research status of TCM as anti-AD drug.

In this review, we show TCM’s active components obvious effect on AD. These components are: monomers, such as safflower yellow (SY), crocin, β-asarone, matrine, linalool, icariin, and extracts like *Dracoephalum moldavica* L. flavonoid, *Dendrobium nobile* Lindl. alkaloids, *Achyranthes bidentata* Blume, and *Coptis chinensis* Franch. watery extract. It is shown that among these TCM that have therapeutic potential for AD, the Lamiaceae family accounts for the highest proportion, and for monomer components, the flavonoids, alkaloids, and polyphenols have significant activity in treating AD. We summarize these in prevention and treatment of AD by reducing Aβ production, apoptosis, autophagy, neuroinflammation, oxidative stress, and mitochondrial dysfunction.

## AD Treatment According to Pathological Processes

**Figure 2:**
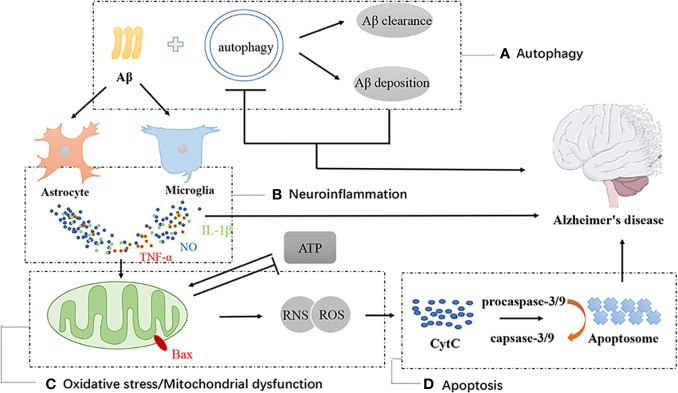
Schematic diagram of autophagy, neuroinflammation, oxidative stress, mitochondrial dysfunction and apoptotic in AD. Autophagy is positive in alleviating AD through promoting Aβ degradation, but hyperactive autophagy is harmful to neuron survival **(A)**. The depositions of Aβ activates the astrocytes and microglia which would secrete oxidative species, such as nitric oxide, and pro-inflammatory cytokines **(B)**. Cytokines on the cell surface and activate pro-apoptotic signaling cascades. Mitochondrial dysfunction cause mitochondria to produce elevated levels of reactive oxygen and nitrogen species (ROS and RNS). Enhancement of ROS and RNS aggravates mitochondrial dysfunction **(C**, **D)**, finally causing release of the pro-apoptotic signaling protein, CytC. CytC contributes to formation of the apoptosome **(D)**. These factors all cause death of neuronal populations and lead to neurodegenerative disease.

According to AD pathogenesis, the effective therapeutic effects of TCM’s monomers and extracts generally exert their effects in the following ways (**Figure 2**).

### β-Amyloid Production Reduction

Aβ plays a crucial role in AD pathogenesis, and neurotoxicity induced by Aβ is the chief cause of AD (Hung and Fu, 2017). Increase in Aβ production and a decrease in Aβ degrading enzymes possibly leads to Aβ aggregation. These leads to NFTs formation and neurodegeneration. APP accumulation causes Aβ deposition, leading to cognitive impairment (Selkoe and Hardy, 2016). Therefore, reducing Aβ deposition is one of the ways to treat AD.

### Anti-Apoptosis Effect

Apoptosis is an active regulation process of cell death, and inducing apoptosis is an important part of Aβ-induced cell toxicity. Three major apoptotic pathways include mitochondrial pathway, endoplasmic reticulum pathway, and death receptors pathway. Mitochondrial pathway occurs mainly through reversing the mitochondrial membrane and the expression of cytochrome c (CytC). Endoplasmic reticulum pathway is mainly caused by Ca^2+^imbalance, which disrupts normal endoplasmic reticulum activities, causing an overload response in the endoplasmic reticulum, triggering the caspase receptor pathway cascade by activating caspase-3 and caspase-9 (Zhang et al., 2014). Generally, apoptosis occurs mainly through the cytochrome c/caspase-9 pathway and the caspase-8 pathway. When the cell receives signals to induce apoptosis, the expression of Bax in the outer membrane increases, accelerating CytC activity, leading to caspase-3 and caspase-9 activation (Nguyen et al., 2013). Apoptosis is a key link in Aβ-induced cytotoxicity, thus anti-apoptosis is recognized as an important way to treat AD.

### Induce or Attenuate Autophagy Effect

Autophagy is a self-degradative process and a pervasive lysosomal degradation pathway to eliminate damaged organelles and proteins common in neurons. The process of autophagy can lead to recycling of cell material and homeostasis preservation (Rami, 2009; Hensley and Harris-White, 2015). The pathways involved in the regulation of autophagy are very complex, including phosphatidylinositol 3-kinase (PI3K)/protein kinase B (AKT)/mechanistic target of rapamycin (mTOR) pathway, AMP-activated protein kinase (AMPK) pathway, renin-angiotensyns-tem (RAS)/cyclic adenosine monophosphate (cAMP)/proteinkinase A (PKA) pathway, p53 pathway, Class I PI3K/PKB pathway, and PI3K III pathway. These pathways are associated with most neurodegenerative diseases including AD. Recent studies suggest that autophagy plays an important role in Aβ clearance. Aβ-containing autophagosomes bind to lysosomes and causes Aβ to degrade during autophagy (Xue et al., 2014). Not only is the APP protein processing related to autophagy, but the clearance of Aβ deposition and maintenance of neuron function is too closely related to autophagy (Pickford et al., 2008).

### Anti-Neuroinflammation

Neuroinflammation has been identified as an important process in the pathogenesis of AD (Latta et al., 2015). Neuroinflammation is closely related to the activation of microglia and astrocytes. Activation of microglia and astrocytes produces a series of pro-inflammatory and cytotoxic factors, such as inducible nitric oxide synthase (iNOS), interleukin-1β (IL-1β), tumor necrosis factor alpha (TNF-α), cyclooxygenase-2 (COX-2), and interleukin-6 (IL-6) (Barger and Harmon, 1997; Wu et al., 2011; Lyman et al., 2014). These cytokines cause neuronal damage and eventually cell death. Overloaded Aβ leads to the activation of astrocytes and microglia and produces these factors. Neural inflammation in AD involves several important signaling pathways such as Toll-like receptor4 (TLR4) pathway and nuclear factor kappa B (NF-κB) pathway. TLR4-mediated signaling pathway comprises of two pathways: MyD88-dependent pathway and TRIF-dependent pathway. Currently, it is believed that proinflammatory cytokines activate microglial cells through TLR4 receptor. NF-κB is a common pathway for multiple signaling pathways that lead to inflammation and also a critical transcription factor involved in AD inflammatory processes (Granic et al., 2009). These suggest that inhibition of neuroinflammation and the NF-κB pathways is an effective way to treat AD.

### Reduce Oxidative Stress

Oxidative stress is another important factor in the AD pathogenesis. TCM containing antioxidants reduces the risk of AD by inhibiting oxidative stress (Min and Min, 2014; Fan et al., 2017). Oxidative stress leads to excess reactive oxygen species (ROS) generation and DNA oxidation, and inhibits antioxidant substances, such as super oxide dismutase (SOD), glutathione peroxidase (GSH-Px). Enhancing ROS and nitrogen species (RNS) aggravates mitochondrial dysfunction and has a detrimental effect on the cellular DNA, proteins, and lipids. Deposited Aβ causes oxidative stress, subsequently increasing ROS levels (Miranda et al., 2000; Butterfield et al., 2006). Consequently, the high level of ROS and RNS lead to release of pro-apoptotic signaling protein, CytC. CytC accelerates the formation of apoptosome, causing neurodegenerative disease (Bhat et al., 2015).

### Reduce Mitochondrial Dysfunction

Mitochondrial dysfunction is another important factor in AD pathogenesis. Extracellular deposits of Aβ can access inside mitochondria and disrupt the normal state of mitochondria, leading to imbalanced mitochondrial membrane potential. The aggregation of Aβ in extracellular are associated with mitochondrial dysfunction and neuronal structural damage. Mitochondrial damage leads to the loss of adenosine triphosphate (ATP) and ROS increase, leading to the cell apoptosis (Reddy and Beal, 2008; Moreira et al., 2010; Onyango et al., 2016).

## TCM for AD Treatment Via Reducing B-Amyloid Production

### Ginseng Protein

Ginseng (*Panax ginseng* C.A. Meyer), which belongs to the Araliaceae family. In China, ginseng has a long history of use to prolong life and soothe the puzzle. It exhibits strong therapeutic effects on cognitive impairments and neurodegeneration (Huang et al., 2019a). Ginsenosides are considered the main bioactive constituent of ginseng, and some studies have reported their neuroprotective effect (Yang et al., 2012). Recent research indicates that ginseng protein (GP) has a very significant neuroprotective effect in the treatment of AD. It inhibits Aβ1-42 and tau pathology, increases the mRNA and protein expression of PI3K, p-Akt/Akt, and Bcl-2 (B-cell lymphoma 2)/Bax (Bcl-2 associated X) in the hippocampus. GP improves the memory capacity and cognitive function by activating PI3K/Akt signaling pathway (Li et al., 2016a).

### The Total Flavonoid Extract from *Dracoephalum moldavica* L.

*Dracoephalum moldavica* L., a member of Lamiaceae family, possesses important medicinal value on refreshing body and mind as well as relieving sore throat and cough. In addition, *D. moldavica* has strong clinical effects on asthma, cardiovascular, and cerebral ischemia (Martínez-Vázquez et al., 2012). The flavonoids in *D. moldavica* have been a hotspot due to their extensive biological applications. The total flavonoid extract from *D. moldavica* (TFDM) mainly include apigenin, luteolin, acacetin, gardenin B, serophulein, salvigenin, isorhamnetin, tilianin, agastachoside, and kaempferol. These extracts are flavone, flavonol, and glycosides. The linked sugars are glucose, xylose, and their derivatives. TFDM are extracted from the aerial part of *D. moldavica* (Liu et al., 2019). A recent research revealed that TFDM treatment improved the memory capacity and inhibited neurodegeneration. TFDM decreased insoluble Aβ levels by reducing Aβ deposition and enhanced the antioxidant defense capacity. TFDM inhibited Aβ production pathway, which is related to the down-regulation of β-secretase and β-C-terminal fragments in the brain of APP/PS1 mice. TFDM treatment activated the nuclear translocation of phospho-extracellular signal-regulated kinase 1/2 (ERK1/2) that led to elevated brain-derived neurotrophic factor (BDNF) levels through enhanced cAMP response element-binding protein (CREB) activation. TFDM can protect injured cells, and is associated with reducing APP and Aβ_1–42_ levels, hence exerts beneficial effects (Liu et al., 2018).

### Safflower Yellow

SY is the main active chalcone glycoside compound extracted from *Carthamus tinctorius* L. (safflower), and belongs to Compositae or Asteraceae family. It is widely used in TCM for management of dysmenorrhea, amenorrhea, and joint pain. Besides, safflower seeds have been reported to attenuate memory impairment (Kim et al., 2019). SY improved cognitive functions and ameliorated the memory loss of APP/PS1 mice. SY decreased the level of Aβ and overactivation of astrocytes in AD rats. Meanwhile, SY increased SOD and GSH-Px levels, and decreased malondialdehyde (MDA) and acetylcholinesterase (AChE) expression in brain tissues of AD mouse model. Moreover, SY was able to inhibit cyclin-dependent kinase 5 (CDK-5) and glycogen synthase kinase-3 (GSK-3) signaling pathways, which are upregulated in AD mouse model (Ma et al., 2015).

### Emodin

Emodin is the main anthraquinone compound from *Rheum officinale* Baill. (Polygonaceae family) is extensively used in TCM and is the monarch drug in some TCM prescriptions with brain protection properties, such as *Tiao-wei-cheng-qi Decoction*, *Da-cheng-qi Decoction* (Gong et al., 2011). Emodin suppresses Aβ deposition and tau phosphorylation. Furthermore, emodin downregulates the activity of β-site APP-cleaving enzyme 1 (BACE1) and increases protein phosphatase 2A levels (Zeng et al., 2019).

### Onjisaponin B

Onjisaponin B is the main active saponin constituent from Radix Polygalae. Radix Polygalae (the root of *Polygala tenuifolia* Willd.) belongs to Polygalaceae family and is a typical herbal medicine and has been extensively used for the treatment of dementia. Radix Polygalae plays an important role in most nootropics prescriptions (Zhang et al., 2008; Wang et al., 2015a; Wang et al., 2015b). Onjisaponin B reduces Aβ production without directly inhibiting of BACE1 and γ-secretase activities, promoting APP degradation (Li et al., 2016b) (**Table 1**).

**Table 1:** TCM for treating AD by reducing β-Amyloid production.

Numbers	Compounds	Chemistry structure	Dosages	Activities	Molecular mechanism	Models	References
1	Ginseng protein	—	0.05–0.1 g/kg twice daily	Improve the memory ability and cognitive and reduce Aβ production	Inhibit Aβ_1-42_ and p-tau and increase the mRNA and PI3K, p-Akt/Akt, and Bcl-2/Bax	d-galactose/AlCl_3_ induced rat model	Li et al., 2016a
2	*Dracoephalum moldavica* L. flavonoid	—	200 mg/kg	Reduce Aβ deposition and insoluble Aβ levels	Attenuate Aβ-induced toxicity through anti-amyloidogenesic and neurotrophic pathways	Heterozygous APPswe/PS1Δ9 transgenic founder mice	Liu et al., 2018
3	Safflower yellow	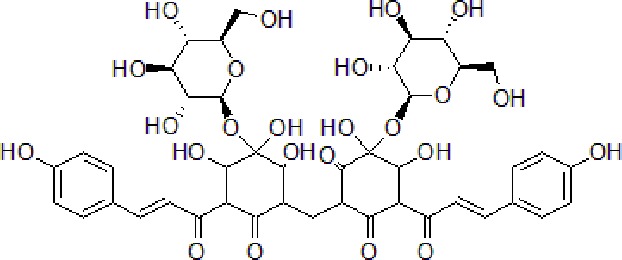	10–30 mg/kg	Improve cognitive function and ameliorate the learning and memory deficits	Decrease Aβ accumulation and overactivation of astrocytes	APP/PS1 transgenic mice	Ma et al., 2015
4	Emodin	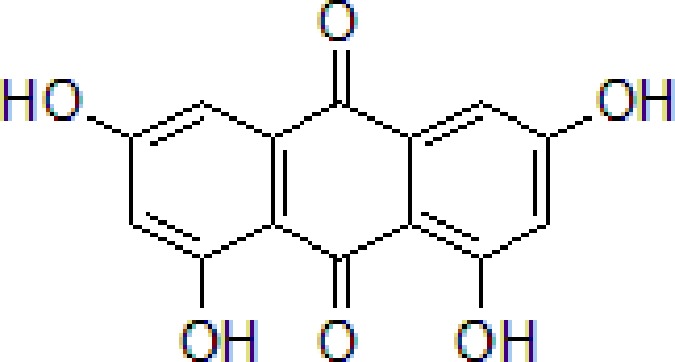	80 mg/kg/day	Improve the memory ability and cognitive, reduce Aβ production	Reduce the levels of Aβ and tau phosphorylation	Hyperhomocysteinemia (HHcy) induced rats	Zeng et al., 2019
5	Onjisaponin B	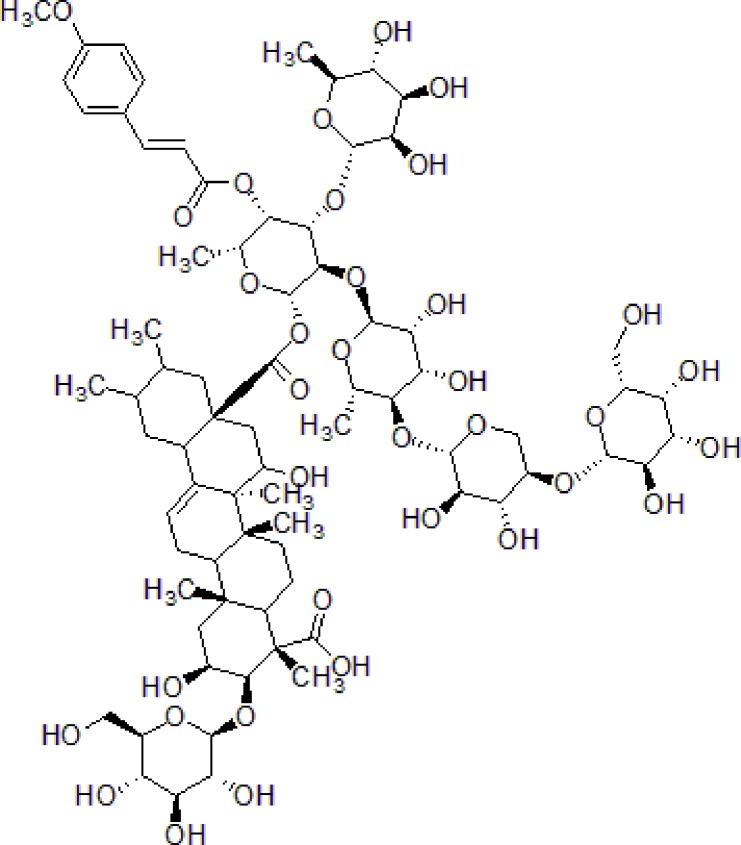	1mg/mL, 200μL	Suppress Aβ production, improve learning and memory capacity	Suppress Aβ production promoted the degradation of APP	The APPswe/PS1ΔE9 (APP/PS1) double-transgenic mice	Li et al., 2016a

## TCM for AD Treatment *Via* Anti-Apoptosis

### Morroniside

Morroniside is a natural iridoid glycoside in *Cornus officinalis* Sieb. et Zucc (Cornaceae), which is widely used in TCM to treat dementia. The dry ripe sarcocarp of *C. officinalis* contains loganin, morroniside, and other bioactive compounds hence has great medicinal value (Wang et al., 2009). Morroniside prevents H_2_O_2_ or Aβ_1-42_-induced apoptosis through some complex apoptotic pathways, including Bcl-2, caspases, and mitochondria-dependent cell death pathways. Morroniside inhibits the expression of Bax, Cytc, cleaves caspase-3, and increases the level of Bcl-2. Morroniside exerts anti-apoptosis effect *via* attenuating c-Jun N-terminal kinase (JNK) and p38 MAPK phosphorylation in Aβ_1-42_-induced PC12 cells (Chen et al., 2018b).

### Curcumin

Curcumin is an active polyphenol extraction isolated from *Curcuma longa* L. It is a member of ginger family and is important TCM with a variety of pharmacological activities, such as anti-inflammatory, anti-cancer, and dementia prevention. Several studies have shown that *C. longa* is a potential neuroprotective drug (Witkin and Li, 2013). Curcumin significantly decreased Aβ-induced cytotoxicity by inhibiting mitochondria-mediated apoptosis *via* regulation of Bcl-2 family. Curcumin inhibited Aβ-induced DNA damage by reducing of ROS generation through p38 MAPK and AKT pathways. Curcumin treatment also protected rat PC12 cells from Aβ_25-35_-induced reduction in cell viability, the level of lactate dehydrogenase (LDH) and MDA. This process was associated with high expression of N-methyl-d-aspartate receptor (NMDAR) and NMDAR subunit 2A (NR2A) (Fan et al., 2017). Furthermore, curcumin was found to protect neuronal cell effectively and attenuate apoptosis by regulating intracellular Ca^2+^ release, ROS, and mitochondrial membrane potential depolarization level in SH-SY5Y cells (Uğuz et al., 2016).

### Triptolide

Triptolide is the main natural diterpene extracted from *Tripterygium wilfordii* Hook F. belonging to Celastraceae family, has been used in TCM for centuries. Recently, several reviews are published on *T. wilfordii* that exhibits therapeutic efficacies in the treatment of neurodegenerative diseases (Schwartz and Deczkowska, 2016). Triptolide remarkably inhibited the neuronal cells apoptosis and increased intracellular Ca^2+^ concentration induced by Aβ and Aβ_25–35_. Triptolide effectively ameliorates Aβ-induced cell injury by suppressing Aβ levels and chemokine receptor 2 (CXCR2) activity (Gu et al., 2004; Wang et al., 2015a).

### Crocin

Crocin is a bioactive carotenoid extracted from *Crocus sativus* L. (Iridaceae family) and is an important TCM known for its medicinal properties in neuropsychiatric disorders, such as depression, seizure, anxiety, and neurodegenerative disease. (Modabbernia and Akhondzadeh, 2013). Crocin can improve memory impairment and learning ability by reducing neuron apoptosis and Bax levels as well as increasing the expression of Bcl-2 in hippocampus and prefrontal cortical neurons (PFC) (Lin et al., 2019a). Aβ administration in hippocampus significantly increases proteins and factors associated with autophagy and apoptosis, such as Beclin-1, LC3-phosphatidylethanolamine conjugate (LC3-II)/cytosolic LC3 (LC3-I) ratio, Bax/Bcl-2 ratio, and cleaved caspase-3 (Asadi et al., 2015). Essentially, crocin alleviates malathion-induced neurological alterations and cognitive impairment by exerting its anti-apoptotic effects (Mohammadzadeh et al., 2019). These show that crocin has potential therapeutic effects on AD (**Table 2**).

**Table 2:** TCM for treating AD by anti-apoptosis.

Numbers	Compounds	Chemistry structure	Dosages	Activities	Molecular mechanism	Models	References
1	Morroniside	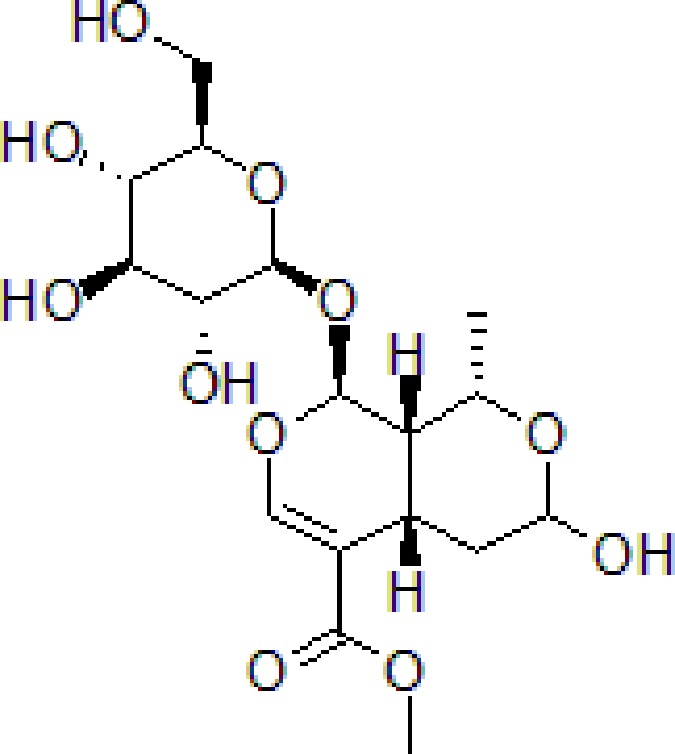	100 μM	Inhibit apoptosis	Inhibit apoptosis *via* attenuating JNK and p38 MAPK phosphorylation	PC12 cells	Chen et al., 2018b
2	Curcumin	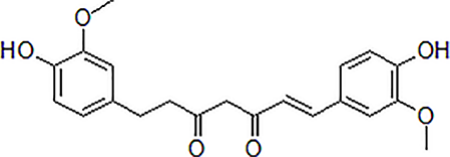	12.5–200 μM	Reversal neurotoxicity, inhibit apoptosis	Inhibit apoptosis though p38 MAPK and AKT pathways	PC12 cells	Uğuz et al., 2016; Fan et al., 2017
3	Triptolide	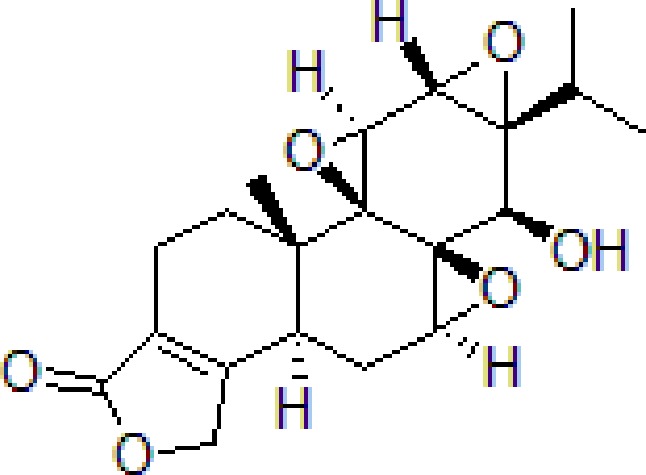	10^−11^ mol/L	Inhibit apoptosis	Inhibit intracellular Ca^2+^ and apoptosis	PC12 cells	Gu et al., 2004; Wang et al., 2015a; Xu et al., 2016
4	Crocin	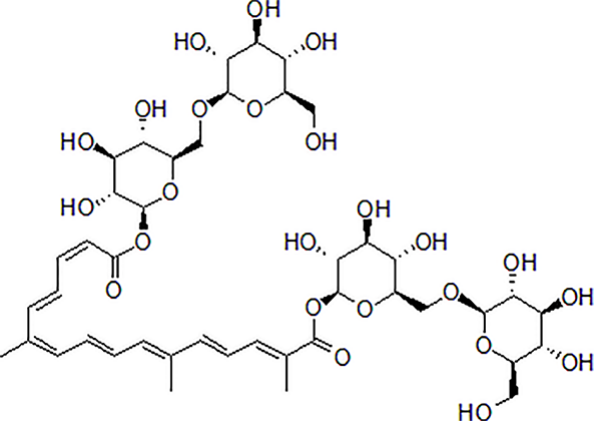	40 mg/kg	Regulate endoplasmic reticulum stress and apoptosis	Increase the autophagy and apoptosis biomarkers	Male Wistar rats	Asadi et al., 2015; Lin et al., 2019a; Lin et al., 2019b; Mohammadzadeh et al., 2019

## TCM for AD Treatment *via* Autophagy Inducing or Attenuation

### Berberine

Ber is an alkaloid extracted from *Coptis chinensis* Franch., a member of Ranunculaceae family extensively used in TCM. *Coptis chinensis* is a major ingredient of *San-Huang-Xie-Xin-Tang*, a traditional Chinese formula, used to manage cardiovascular and neurodegenerative diseases (Li et al., 2018). Ber improves memory retention and spatial learning capacity by promoting Aβ clearance. It exerts neuroprotective effect by facilitating autophagy through LC3-II, Beclin-1, hVps34, sequestosome 1 (p62), and Cathepsin-D activities as well as Bcl-2 brain levels reduction (Huang et al., 2017; Zhang et al., 2018).

### Andrographolide

Andrographolide (Andro) is the main bioactive labdane diterpenoid component of *Andrographis paniculata* (Burm.f.) Nees, a typical TCM of Acanthaceae family. Recently, extensive researches have shown that *A. paniculate* can ameliorate cognitive impairment (Serrano et al., 2014; Thakur et al., 2016; Geng et al., 2018). Also, Andro can ameliorate cell viability and normalize the abnormal nuclear morphology by inhibiting Aβ_1–42_-induced abnormal changes in LDH, MDA, ROS, and mitochondrial membrane potential. Andro significantly improves Aβ_1–42_-induced cognitive impairment and learning function by activating autophagy-related genes and proteins, such as Beclin-1, LC3 nuclear factor, E2-related factor 2 (Nrf2), p62, and p21. In addition, Andro can exert autophagy effect *via* Nrf2-mediated p62 signaling pathway (Gu et al., 2018).

### Geniposide

Geniposide is an iridoid glycoside component of *Gardenia jasminoides* Ellis. and is an essential component of traditional phytomedicines (Shan et al., 2017). It belonging to Rubiaceae family, and is widely used in TCM. Geniposide exhibits significant therapeutic effect in the treatment of brain disorders and neurodegenerative disease (Zhao et al., 2016). Geniposide improved cognitive function by decreasing Aβ_1-40_ deposition in the brain tissue of AD mouse model by activating glucagon-like peptide-1 (GLP-1) receptors, which regulates mTOR. Thus, down-regulating mTOR signaling leading to enhanced cellular autophagy and increased lysosomal clearance of Aβ fibrils. Geniposide increased p-Akt/Akt, p-mTOR/mTOR, expression of LC3-II and Beclin1. This indicates that geniposide has a therapeutic effect on the AD neuropathological damage (Zhang et al., 2019).

### β-Asarone

β-asarone is the major component of *Acorus tatarinowii* Schott (Wu and Fang, 2004). *Acorus tatarinowii*, belongs to Acoraceae family, is commonly used as TCM. It is known for a variety of pharmacological activities, specifically in treatment of neurodegenerative diseases (Li et al., 2010). β-asarone increases cell viability by decreasing neuron specific enolase (NSE) levels and Beclin-1 in addition to Aβ_1–42_-induced autophagy attenuation through Akt-mTOR signaling pathway (Xue et al., 2014).

### Dendrobium nobile Lindl Alkaloids

*Dendrobium nobile* Lindl. has been used for medicinal purpose for centuries and is a member of Orchidaceae family. It has been widely used for kidney injury treatment. Alkaloids are considered as the main characteristic potent ingredients of *D. nobile* (Ng et al., 2012). *Dendrobium nobile* Lindl alkaloids (DNLA) is an active alkaloid from *D. nobile* Lindl. and mainly includes dendrobine, dendroxine, nobiline, dendrine, 6-hydroxy-dendroxine, *N*-methyl-dendrobine, and *N*-isopentenyl-dendrobine (Inubushi et al., 1964; Granelli et al., 1970). DNLA are able to increase autophagic flux, inhibit axonal degeneration by increasing Beclin-1 expression. DNLA increases autophagosome formation and autophagosome-lysosome fusion in hippocampus region (Li et al., 2017).

### Euxanthone

Euxanthone is a xanthone derivative extracted from *Polygala caudata* Rehder & E.H.Wilson mainly distributed in southwestern China that has been extensively used in TCM (Pan and Mao, 1984). uxanthone can attenuate memory impairment and learning dysfunction by reversing Aβ_1-42_-induced neuronal apoptosis and autophagy. Euxanthone protected PC12 cells against Aβ_1-42_-induced oxidative stress and apoptosis by inducing autophagy *via* LC3B-II enhancement and p62degradation (Yuan et al., 2018) (**Table 3**).

**Table 3:** TCM for treating AD by inducing or attenuating autophagy effect.

Numbers	Compounds	Chemistry structure	Dosages	Activities	Molecular mechanism	Models	References
1	Berberine	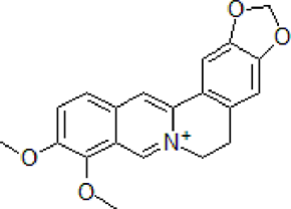	50–100 mg/kg/day	Promote Aβ clearance, improve learning and memory capacity	Induce autophagy by activating Bcl2/Beclin1 signaling	3 × Tg-AD mice	Huang et al., 2017; Zhang et al., 2018
2	Andrographolide	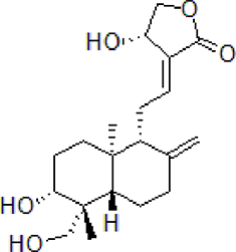	20 μM	Decrease cell death	Induce autophagy through activation of the Nrf2-mediated p62 signaling pathway	PC12 cells	Gu et al., 2018
3	Geniposide	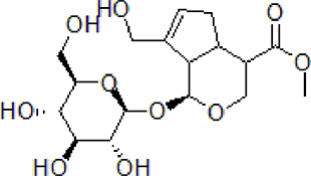	50 mg/kg/day	Reduce Aβ_1–40_ level, promote Aβ clearance, improve cognitive function	Induce autophagy by down regulation of mTOR	Age-matched C57BL/6 wild-type (WT) mice or APP/PS1 mice	Shan et al., 2017; Zhang et al., 2019
4	β-asarone	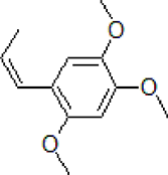	240 μg/mL	Increase cell viability, decrease NSE levels	Attenuate autophagy by Akt-mTOR signaling pathway	PC12 cells	Wu and Fang, 2004; Xue et al., 2014
5	Dendrobium nobile Lindl. alkaloids	—	3.5–350 ng/mL	Inhibit axonal degeneration	Induce autophagy by promoting Beclin1	Aβ_25–35_-induced hippocampus primary neuron	Li et al., 2017
6	Euxanthone	—	30 mg/kg or 60 mg/kg	Attenuate memory and spatial learning dysfunction	Induce autophagy by increasing the expression level of LC3B-II and enhancing the degradation of p62	Sprague-Dawley rats	Pan and Mao, 1984; Yuan et al., 2018

## TCM for AD Treatment *Via* Anti-Neuroinflammation

### Gypenoside

Gypenoside is the major active saponin ingredient of *Gynostemma pentaphyllum* (Thunb.) Makino, a member of Cucurbitaceae family known for strengthening effect on the heart and brain (Lin et al., 1993). Recent researches have shown that Gypenoside has a potential in the treatment of neurodegenerative diseases. The deposition of Aβ activated microglia and astrocyte, leading to the production of inflammatory factors, IL-1β, IL-6, and TNF-α which caused neuronal death (Kumar et al., 2018; Seo et al., 2018). Gypenoside attenuates Aβ-induced inflammation by downregulating the release of proinflammatory factors such as iNOS, TNF-α, IL-1, and IL-6, as well as increasing anti-inflammatory factors release, such as IL-10. Gypenoside can increase the levels of arginase-1 (Arg-1) protein, brain-derived neurotrophic factor (BDNF), and glial cell-derived neurotrophic factor (GDNF) secretions. The process is mediated by suppressing cell signaling protein 1 (SOCS1) (Cai et al., 2016).

### Steroid-Enriched Fraction of *Achyranthes bidentata* Blume (ABS)

*Achyranthes bidentata* Blume (Amaranthaceae family) is commonly used in the treatment of dementia in TCM. The roots of *A. bidentata* are rich in pharmacological active ingredients (He et al., 2017). Recent reports have shown that ABS can attenuate cognitive dysfunction and neuroinflammation. ABS alone (50 mg/kg) reduced the levels of TNF-α in brain and decreased neuroinflammation by modulating ERK and NF-κB pathway (He et al., 2014; Xu X. X. et al., 2017; Lin et al., 2019b).

### Matrine

Matrine is the major bioactive alkaloid of the *Sophora flavescens* Ait. (Fabaceae family), a famous traditional Chinese herbal medicine used to treat dementia. Matrine can improve cognitive deficits and learning ability. Matrine attenuated Aβ_42_-induced memory deficits, cytotoxicity, and the formation of senile plaques in AD transgenic mice. This was through reducing Aβ deposition and proinflammatory cytokines *via* glycation end products (RAGE) signaling pathway (Cui et al., 2017).

### Loganin

Loganin is also a natural iridoid glycoside from *Cornus officinalis* Sieb. Loganin can attenuate Aβ- induced microglia activation, over-production of TLR4, Myeloid differentiation factor 88(MyD88), TNF receptor-associated factor 6 (TRAF6). The anti-inflammation effect of loganin is *via* inhibiting elevation of TNF-α, IL-6, macrophage chemotactic protein 1 (MCP-1), NO, prostaglandin E2 (PGE2), and up-regulating iNOS and COX-2 (Cui et al., 2018).

### Scutellarein

Scutellarein is a natural ingredient extracted from *Scutellaria baicalensis* Georgi., and belongs to Lamiaceae family. It has been traditionally used to treat various diseases, including dementia. Recent reports revealed that flavonoids from *S. baicalensis*, such as baicalin, baicalein, and scutellarin, exert neuroprotective effects (Jin et al., 2019). Scutellarein, a hydrolysate of scutellarin, can also decrease hippocampal Aβ and MDA content while increasing superoxide dismutase (SOD) and acetylcholine (Ach) expression. Moreover, it has a protective effect on Aβ-exposed apoptosis, counteracts the Aβ-induced Bcl-2 expression decrease and inhibits the expression of Bax and cleaved caspase3. In PC12 cells, scutellarein attenuated Aβ-induced cell death, cognitive impairment, hippocampal alterations, hippocampal neuroinflammation, and NF-κB activation. In summary, scutellarein inhibited Aβ−induced PC12 cell apoptosis. This demonstrated that scutellarein has potential therapeutic application in AD (Huang et al., 2019b).

### Oridonin

Oridonin (Ori) is the main active diterpenoid of *Isodon rubescens* (Hemsl.) H. Hara (syn. *Rabdosia rubescens* Hara) of Lamiaceae family and has been used as Chinese herbal medicine due to its biological activities (Zhang et al., 2004). Ori has been shown to attenuate memory impairment and has anti-inflammatory effects. Ori exerts anti-inflammatory effects by inhibiting the activation of glial, decreasing the release of inflammatory cytokines IL-1β, IL-6, and TNF-α. This process is potentially associated with the NF-κB pathway inhibition (Wang et al., 2014).

### Hydroxy-Safflor Yellow A

Hydroxy-safflor yellow A (HSYA) is active chalcone glycoside extracted from *Carthamus tinctorius* L.(Sun et al., 2010). HSYA can ameliorate Aβ_1–42_-induced memory deficiency and microglia and astrocytes activation by downregulating the mRNA expression of pro-inflammatory cytokines. HSYA up-regulated the janus kinase-2 (JAK2) signal transducers and activators of transcription 3 (STAT3) pathway and inhibited the activation of NF-κB signaling pathways. HSYA protects Aβ_1–42_-induced AD model mice through JAK2/STAT3/NF-κB pathway (Zhang et al., 2014).

### Diammonium Glycyrrhizinate

Diammonium glycyrrhizinate (DG) is the most important active ingredient of *Glycyrrhiza uralensis* Fisch. ex DC. of the Fabaceae family. It has been widely used in TCM to treat variety of diseases. Recent pharmacological reports revealed that *G. uralensis* exerts neuroprotective effect in AD (Ahn et al., 2006). DG attenuated Aβ_1–42_-induced memory impairment and activation of microglia and inflammation in AD mice model by inhibiting the activation of MAPK and NF-κB signaling pathways (Zhao et al., 2013) (**Table 4**).

**Table 4:** TCM for treating AD by anti-neuroinflammation.

Numbers	Compounds	Chemistry structure	Dosages	Activities	Molecular mechanism	Models	References
1	Gypenoside	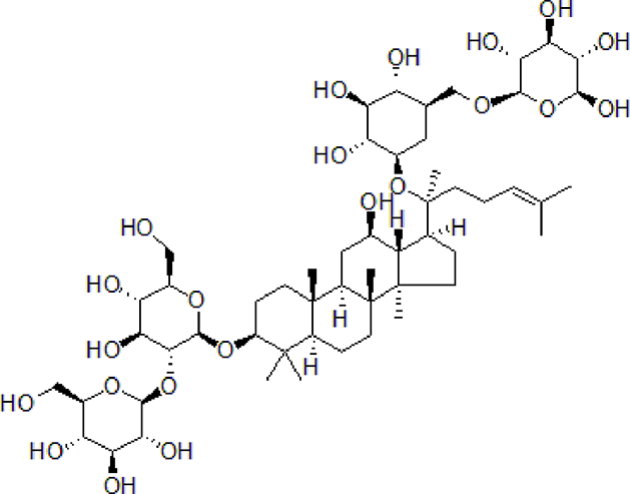	50 μg/mL	Attenuate inflammation	Attenuate Aβ induced inflammation *via* SOCS1 Signaling	N9 microglial cells	Cai et al., 2016; Kumar et al., 2018; Seo et al., 2018
2	Achyranthes bidentata Blume	—	50 mg/kg	Improve cognitive function, decrease neuroinflammation	Decrease oxidative stress and neuroinflammation through modulating ERK pathway, NF-κB phosphorylation, and translocation	Male Sprague-Dawley rats	Lin et al., 2019b
3	Matrine	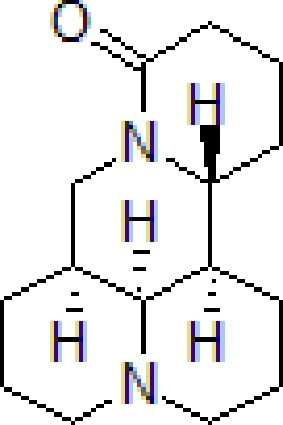	10–50 μM	Improve cognitive deficits and learning ability	Decrease neuroinflammation though Aβ/RAGE signaling pathway	SH-SY5Y cells	Cui et al., 2017
4	Loganin	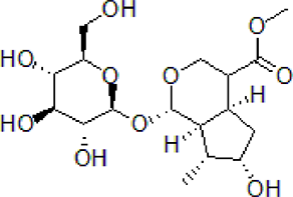	10–30 μM	Decrease neuroinflammation	Decrease neuroinflammation *via* regulating TLR4/TRAF6/NF-κB axis	BV-2 microglia cells	Cui et al., 2018
5	Scutellarein	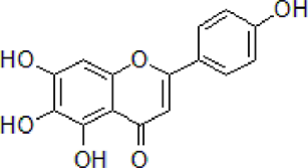	50 mg/kg, intraperitoneally	Suppress neuroinflammation	Increase Bcl-2 and suppress Beclin-1 expression *via* inhibition of the NF-κB pathway	PC12 cells, male Wistar rats	Huang et al., 2019b
6	Oridonin	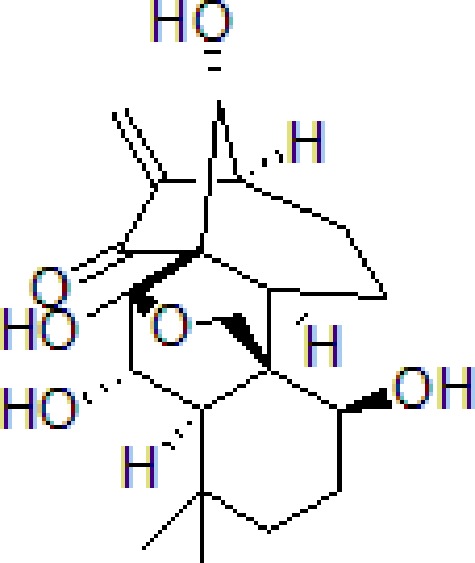	10 mg/kg/day, i.p. for 15 days	Inhibit glial activation, decrease the release of inflammatory cytokines and attenuate memory deficits	Attenuate Aβ_1–42_-induced neuroinflammation and inhibits NF-κB pathway	Male C57BL/6 (B6) mice	Wang et al., 2014
7	Hydroxy-safflor yellow A	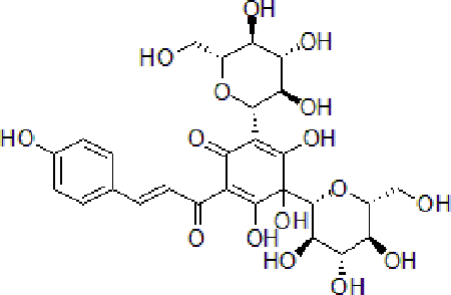	20 mg/kg per day, i.p.	Ameliorate the memory deficits and decrease the mRNA expression of pro-inflammatory mediators	Attenuate Aβ_1-42_-induced inflammation by modulating the JAK2/STAT3/NF-κB pathway	Male ICR mice	Sun et al., 2010; Zhang et al., 2014
8	Diammonium Glycyrrhizinate	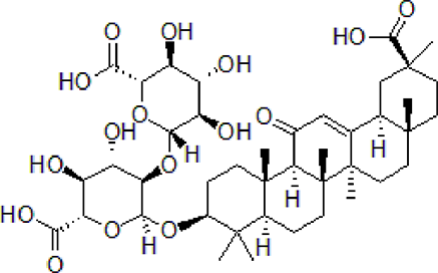	10 mg/kg per day, i.p.	Attenuate the memory deficits and suppress Aβ_1–42_-induced activation of microglia and inflammation	Attenuate Aβ_1–42_-induced neuroinflammation and regulate MAPK and NF-κB pathways	SH-SY5Y and HT-22 cells or male ICR mice	Zhao et al., 2013

## TCM for AD Treatment Via Reducing Oxidative Stress

### Schisanhenol

Schisanhenol is a lignan compound from *Schisandra sphenanthera* Rehder & E. H. Wilson of Schisandraceae family. It has been used extensively in TCM to treat age-related diseases (Hung et al., 2007). Schisanhenol can remove oxygen radicals and inhibit lipid peroxidation and apoptotic cell death induced by oxidative stress. In addition, schisanhenol can improve learning and memory as well as attenuate oxidative damage by reducing the activity of AChE *via* sirtuin 1 (SIRT1)- coactivator 1-α (PGC-1α)-Tau signaling pathway (Yu and Liu, 2008; Han et al., 2019).

### Amentoflavone (AF)

AF is the major active flavonoid found in *Selaginella tamariscina* (P. Beauv.) Spring. This plant belongs to *Schisandraceae* family which has been found exert pleiotropic bioactivities. Recent pharmacological reports provide evidence that *S. tamariscina* could be an effective neuroprotective agent (Shin et al., 2006). It was found that AF decreased Aβ-induced learning and memory deficits and prevented neuronal cell apoptosis, oxidative stress in the hippocampus of rat model. AF increased Nrf2 expression and promoted AMPK signaling. The neuroprotective effect of AF against Aβ_1–42_-induced neurotoxicity is mediated by Nrf2 antioxidant pathways and AMPK signaling (Chen et al., 2018a).

### *Coptis chinensis* Franch. Rhizome Watery Extract

Studies have reported that dried rhizome of *Coptis chinensis* Franch. possess pharmacologically active ingredients. Key among them include Ber, palmatine, coptisine, epiberberine, columbamine, and jatrorrhizine (Luo et al., 2011). Previously, extracts from *Coptis chinensis* Franch. rhizome watery extract (CRE) improved t-BOOH-induced cytotoxicity and cell injury by attenuating mitochondrial membrane depolarization, regulating Thioredoxin-interacting protein (TXNIP) and mitochondrially encoded NADH dehydrogenase. It also conferred neuroprotection by downregulating TXNIP levels (Friedemann et al., 2014).

### Oxymatrine

Oxymatrine (OMT) is a bioactive quinolizidine alkaloid isolated from *Sophora flavescens* Aiton and *Sophora alopecuroides* L. (Qian et al., 2018). A study found that OMT significantly increased cell viability and the cognitive ability of rats. Moreover, treatment with OMT increased the ratio of Bcl-2/Bax and decreased the expression of caspase-3. The protective effect of OMT against Aβ_1-42_-induced neuronal toxicity was linked to the inhibition of MAPK and NF-κB signal pathways (Dong et al., 2019).

### Shikonin

Shikonin is an active naphthoquinone ingredient found in *Lithospermum erythrorhizon* Siebold & Zucc., one of the most important Chinese herbal medicine belonging to the Boraginaceae family. This compound has long been used to treat allergic diseases. Recent reports have provided evidence that roots of *L. erythrorhizon* contain compounds with antioxidant activities (Papageorgiou et al., 2008; Yoshida et al., 2014). Shikonin markedly increased cell viability by upregulating the activity of SOD catalase and GSH-Px. It also decreased levels of MDA and ROS, and stabilized the mitochondrial membrane potential in Aβ_1–42_-treated PC12 cells. The neuroprotective effect of shikonin against Aβ-induced cell damage was due to inhibition of cleaved caspase-3 activity and reduction in ratio of Bcl-2/Bax all of which prevent antioxidants and apoptosis (Tong et al., 2018).

### Linalool (LI)

LI is the main volatile monoterpene compound found in several aromatic plants, such as *Lavandula angustifolia* Mill., *Rosmarinus officinalis* L. and *Coriandrum sativum* L. (Kuroda et al., 2005; Batista et al., 2010; Gastón et al., 2016). In a previous study, LI significantly improved the cognitive performance and alleviated Aβ_1-40_-induced cell injury by increasing the levels of oxidative stress indicators such as SOD, GPX, and AChE. LI decreased the activity of cleaved caspase-3 and caspase-9 and increased the expression level of Nrf2 and heme oxygenase-1 (HO-1). Thus, LI confers neuroprotection by preventing apoptosis, oxidative stress, and activation of Nrf2/HO-1 signaling pathway (Xu P. et al., 2017).

### Schisandrin C

Schisandrin C (SCH-C) is the main antioxidative lignan extracted from *Schisandra chinensis* (Trucz.) Baill. It attenuated impaired cognitive and learning ability by decreasing the activity of total cholinesterase (ChEtotal), significantly increasing the activities of SOD and GSH-Px glutathione. Thus, neuroprotective effect of SCH-C against Aβ_1–42_-induced cell injury is mediated by inhibition of ChEtotal and upregulation of the level of SOD and GSH-Px glutathione (Mao et al., 2015).

### Acteoside

Acteoside, a phenylethanoid glycoside, is isolated from *Cistanche deserticoLa* Y. C. Ma. that belongs to Drobanchaceae family. It has been used to treat age-related disorders and improve kidney function (Jiang and Tu, 2009). Acteoside improved the cognitive function following treatment with d-galactose (d-gal) and AlCl_3_ by increasing the number of neurons and decreasing the level of nitric oxide (NO), nitric oxide synthase (NOS) and caspase-3 protein expression in hippocampal tissues. The mechanisms associated include suppression of oxidative stress and hence neuronal apoptosis (Peng et al., 2015).

### Vanillic Acid

Vanillic acid (VA) is a natural benzoic acid derivative obtained from *Angelica sinensis* (Oliv.) Diels. which belongs to Apiaceae family. It has been used in TCM as a flavoring agent and to treat various diseases. Recent studies have provided evidence that Danggui Buxue Tang (derived from *A. sinensis* (Oliv.) Diels) has the potential to treat AD (Gong et al., 2019). VA significantly improved memory function by decreasing AChE, corticosterone, TNF-α, and inhibiting oxidative stress (Singh et al., 2015).

### Protosappanin A

Protosappanin A (PTA) is the major biphenyl compound extracted from *Caesalpinia sappan* L. (Lignum Sappan), a member of Fabaceae family. This plant has traditionally been used to treat various diseases. Numerous lines of evidence also show that compounds found in *Biancaea sappan* (L.) Tod. (syn. *Caesalpinia sappan* L.) have antioxidant activities (Sasaki et al., 2007). Activation of microglia increases the level of ROS and NO, leading to neuronal and cell injury. PTA exerted immunosuppressive effects and anti-oxidative activities against lipopolysaccharide (LPS)-induced injury on BV-2 microglia by inhibiting the activation of microglia and suppressing ROS and NO levels. Moreover, PTA modulated the CD14/TLR4-dependent IκB-kinase (IKK)/nuclear factor κB (IκB)/NF-κB inflammation signaling pathway which decreased the expression of NADPH oxidase and iNOS (Zeng et al., 2012).

### Salidroside

Salidroside is the main tyrosol-glucoside compound extracted from *Rhodiola rosea* L. that belongs to Crassulaceae family. Recently, it was reported that *R. rosea* contain various compounds such as salidroside with pharmacological properties, including anti-oxidative effects (Kanupriya et al., 2005). Salidroside confers neuroprotection by inducing thioredoxin (Trx), HO-1, and peroxiredoxin-I (PrxI). In addition, it suppresses Aβ_25–35_-induced neuronal injury be decreasing ROS production and restoring mitochondrial membrane potential (MMP) through JNK and p38 MAPK signaling pathways (Zhang et al., 2010) (**Table 5**).

**Table 5:** TCM for treating AD by reducing oxidative stress.

Numbers	Compounds	Chemistry structure	Dosages	Activities	Molecular mechanism	Models	References
1	Schisanhenol	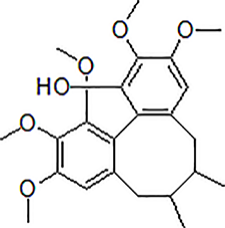	10–100 mg/kg	Improve learning memory and attenuate oxidative damage	Reduce acetylcholinesterase activity and attenuate oxidative damage through SIRT1-PGC-1α-Tau signaling pathway	Scopolamine-treated Kunming mice	Han et al., 2019
2	Amentoflavone	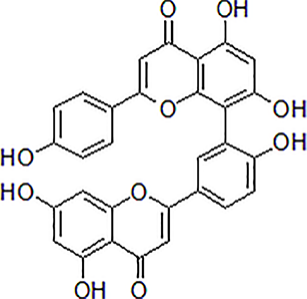	80 mg/kg	Ameliorate memory deficits and oxidative stress	Ameliorate memory deficits and oxidative stress by inducing Nrf2 antioxidant pathways *via* AMPK signaling activation	PC12 cells and rat	Chen et al., 2018a
3	Coptis chinensis Franch. watery extract	—	100 mg/mL	Neuroprotective and against oxidative stress	Have neuroprotective and against oxidative stress and down regulation of TXNIP	SHSY5Y cells	Thomas Friedemann et al., 2014
4	Oxymatrine	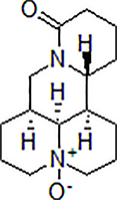	30–120 mg/kg	Increase cell viability and SOD activity	Improve the cognitive ability of rats and has a protective effect on Aβ_1-42_-induced primary neuronal cell by inhibiting MAPK and NF-κB signal pathways	Sprague-Dawley rats	Qian et al., 2018; Dong et al., 2019
5	Shikonin	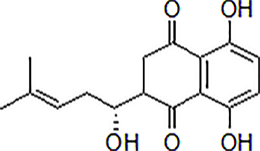	3.47, 10.42, 34.72 µM	Improve cell viability, decrease the MDA and ROS content, and stabilize the mitochondrial membrane potential	Reduce the activity of caspase-3 and moderate the ratio of Bcl-2/Bax through antioxidant and antiapoptotic activities	PC12 cells	Tong et al., 2018
6	Linalool	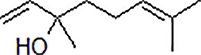	100 mg/kg per day, i.p.	Improve the cognitive performance and reverse the Aβ_1-40_ induced hippocampal cell injury, apoptosis and changes of oxidative stress indicators	Alleviate apoptosis, oxidative stress *via* activation of Nrf2/HO-1 signaling	C57BL/6J mice	Kuroda et al., 2005; Batista et al., 2010; Gastón et al., 2016; Xu P. et al., 2017
7	Schisandrin C	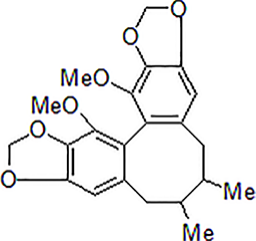	150 μg/kg, 10 mL/kg, injection	Improve the cognitive function and working memory	Inhibit ChEtotal, increased SOD and GSH-px, GSH	Male Kunming mice	Mao et al., 2015
8	Acteoside	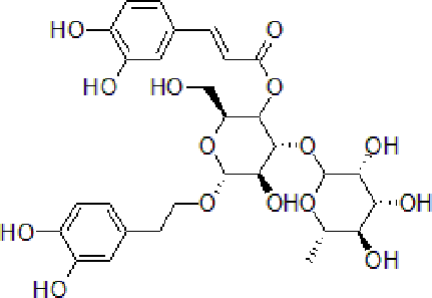	30, 60, and 120 mg/kg/day, for 30 days	Attenuate cognitive impairment and increase the numbers of neurons and Nissl bodies	Decrease the content of NO, the activity of NOS and the expression of caspase-3 protein due to oxidative stress	Kunming mice	Peng et al., 2015
9	Vanillic acid	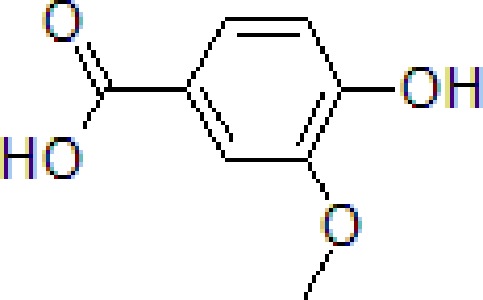	50 and 100 mg/kg	Improve spatial learning and memory	Improve the habituation memory, decrease the AChE, corticosterone, TNF-α by preventing oxidative stress	Swiss albino male mice	Singh et al., 2015
10	Protosappanin A	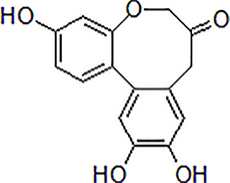	10 mg/kg, 5–50 μM	Inhibit ROS and NO production by suppression of NADPH oxidase and iNOS activity	Modulate IKK/IκB/NF-κB inflammation signal pathway to inhibit the activity and expressions of NADPH oxidase and iNOS	BV-2 cells or ICR mice	ZeNg et al., 2012
11	Salidroside	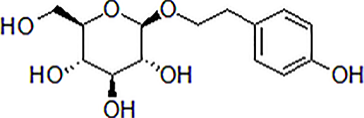	10, 50, 100 mM	Protect neurons from oxidative stress *via* the induction of antioxidant enzymes, Trx, HO-1, and PrxI	Inhibit Aβ_25–35_-induced phosphorylation of JNK and p38 MAP kinase and oxidative stress.	SH-SY5Y cells	Zhang et al., 2010

## TCM Improves AD by Correcting Mitochondrial Dysfunction

### Icariin

Icariin is the principal flavonoid in *Epimedium brevicornu* Maxim. This plant is a member of the *Berberidaceae* family commonly used to formulate Chinese herbal drugs for various diseases. Accumulating evidence demonstrates that *E. brevicornu* confer beneficial effects on the nervous systems (Ma et al., 2011). Icariin administration effectively decreased Aβ deposition in mitochondria and reversed the decline in levels of pyruvate dehydrogenase component α (PDHE1α) and cytochrome c oxidase IV (COX IV) in 3×Tg-AD mice. In summary, Icariin improves cognitive functions and neuronal cell activity, enhancing electron transport chain, N-acetylaspartate level, and brain ATP metabolism (Lustbader et al., 2004; Reddy and Beal, 2008; Chen et al., 2016).

### Salvianolic Acid B

Salvianolic acid B (SalB) is the main active polyphenol found in S*alvia miltiorrhiza* Bge. a member of *Lamiaceae* family. Epidemiological evidence demonstrates that the roots of *S. miltiorrhiza* contain pharmacologically active ingredients with curative potential for various diseases, including AD (Wang et al., 2016). It was previously found that SalB alleviated Aβ-induced increase in glutathione (GSH) activity, lipid oxidation, axonal mitochondrial fragmentation, stabilized mitochondrial membrane potential, improved ATP production, and activity of CytC oxidase, all of which prevented cytotoxicity. Administration of SalB restores the synaptic density of Aβ-treated neurons (He et al., 2018).

### Ligustilide

Ligustilide (LIG) is the main bioactive lipophilic component of Radix angelicae sinensis, *Angelica sinensis* (Oliv.) Diels. LIG reduces Aβ expression and that of dynamin-related protein 1 (Drp1) and increases levels of mitofusin 1 (Mfn1), mitofusin 2 (Mfn2), and Optic atrophy 1(Opa1) in APP/PS1 transgenic mice. LIG exerts anti-oxidant effects by decreasing MDA and ROS levels while increasing the level of Mn-SOD. It also improves memory deficits and mitochondrial function in APP/PS1 mice by reducing Aβ levels, enhancing mitochondrial motility, and restoring synaptic structure (Xu et al., 2018).

### Tetrahydroxy Stilbene Glycoside

Tetrahydroxy stilbene glycoside (TSG) is one of the active polyhydroxystibene component isolated from *Reynoutria multiflora* (Thunb.) Moldenke (syn. *Polygonum multiflorum*), a member of the *Polygonaceae* family. It has been used to treat liver and kidney injury and as an antiaging agent for centuries. Recent reports show that TSG improves cognitive deficits in AD (Um et al., 2006). It also alleviates cell oxidative stress injury by attenuating LDH release, ROS production, and MDA leakage. TSG canceled Aβ-induced loss of MMP, alleviated the release of CytC from mitochondria to cytosol. It also increases the activity of caspase-3 and Bax expression, whereas it decreases Bcl-2 expression. TSG prevents neuronal cell injury by stabilizing mitochondrial function *via* Nrf2-HO-1 pathway (Jiao et al., 2017).

### Hopeahainol A

Hopeahainol A (Hop A) is a potential AChE inhibitor and anti-oxidative polyphenol isolated from *Hopea hainanensis* Merrill & Chun. A previous study revealed that Hop A not only suppressed Aβ levels, but also inhibited the interaction between Aβ1-42 and Aβ-bound alcohol dehydrogenase (ABAD), which partially improved mitochondrial function and oxidative damage. These results reveal that Hop A balances synaptic function and improves memory deficits in APP/PS1 mice. (Zhu et al., 2013) (**Table 6**).

**Table 6:** TCM for treating AD by reducing mitochondrial dysfunction.

Numbers	Compounds	Chemistry structure	Dosages	Activities	Molecular mechanism	Models	References
1	Icariin	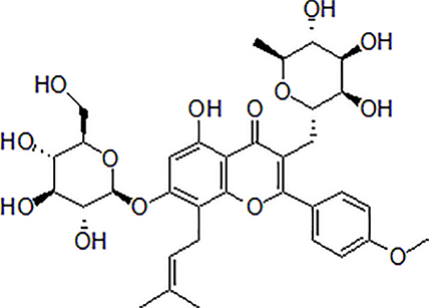	65 mg/kg, 20 μmol/kg/day	Improve spatial learning and memory retention	Ameliorate Aβ elevation in mitochondria and regulate the activity and expression of key mitochondrial enzymes	3 × Tg-AD mice	Lustbader et al., 2004; Reddy and Beal, 2008; Chen et al., 2016
2	Salvianolic acid B	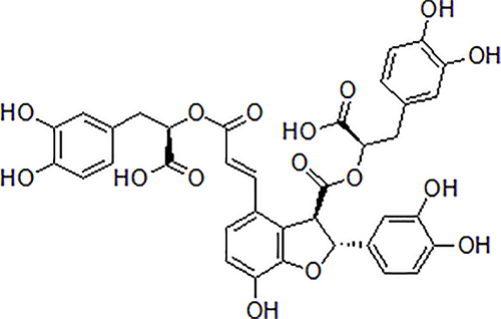	50 μM	Inhibit axonal mitochondrial fragmentation	Attenuate axonal mitochondrial fragmentation and increase kinesin-like protein 1 phosphorylation and restore the synaptic density	Primary cultured mouse neurons cell	He et al., 2018
3	Ligustilide	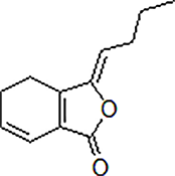	10 or 40 mg/kg	Reduce the level of Drp1 and increase levels of Mfn1, Mfn2, and Opa1	Exerts an antioxidation effect *via* reducing the levels of MDA and ROS and increasing the activity of Mn-SOD	The APPswe/PS1dE9 (APP/PS1) transgenic mouse	Xu et al., 2018
4	Tetrahydroxy stilbene glycoside	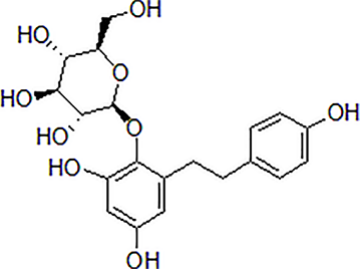	100 μg/mL, 5, 10, 30, 60, 90 μmol/L	Alleviate cell oxidative stress injury and mitochondrial membrane potential	Restore Aβ-induced hippocampal neuronal cell damage by restoring mitochondrial function *via* Nrf2-HO-1 pathway	HT-22 cell	Jiao et al., 2017
5	Hopeahainol A	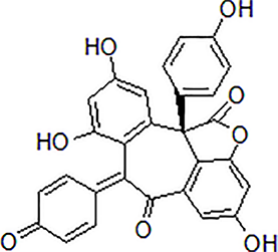	4 mg/kg/day	Attenuate memory deficits	Reduce mitochondrial dysfunction and oxidative stress	APP/PS1 transgenic mice	Zhu et al., 2013

## TCM for AD Treatment Through Various Mechanisms

### Osthole

Osthole, a derivative of coumarin extracted from *Cnidium monnieri (L.) Cusson*, a member of Apiaceae family. Osthole up-regulates miR-107 and inhibits cleaving enzyme 1. It also increases cell survival, reduces LDH leakage. Osthole decreased Aβ levels by up-regulating miR-107 (Jiao et al., 2016).

### *Dendrobium officinale* Polysaccharides

*Dendrobium officinale* Kimura & Migo is one of the most important TCM used to treat age-related disorders. *Dendrobium officinale* polysaccharides (DOP) remarkably attenuated cognitive decline and activated hippocampal microglial by downregulating IL-1β, TNF-α, and IL-6 while upregulating interleukin-10 (IL-10), neprilysin (NEP), and insulin-degrading enzyme (IDE) (Feng et al., 2019) (**Table 7**).

**Table 7:** TCM for treating AD by other ways.

Numbers	Compounds	Chemistry structure	Dosages	Activities	Molecular mechanism	Models	References
1	Osthole	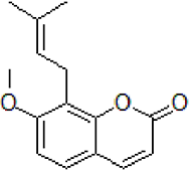	10–100 μM	Decrease Aβ levels	Decrease Aβ levels though up-regulation of miR-107	APP/PS1 transgenic mice and SH-SY5Y cells, HEK293 cell	Jiao et al., 2016
2	Dendrobium officinale polysaccharides	—	5–10 μg/mL	Attenuate cognitive impairment	Attenuate cognitive impairment *via* modulation of microglial activation	BV2 cells, SAMP8 mice	Feng et al., 2019

## Conclusions

**Figure 3:**
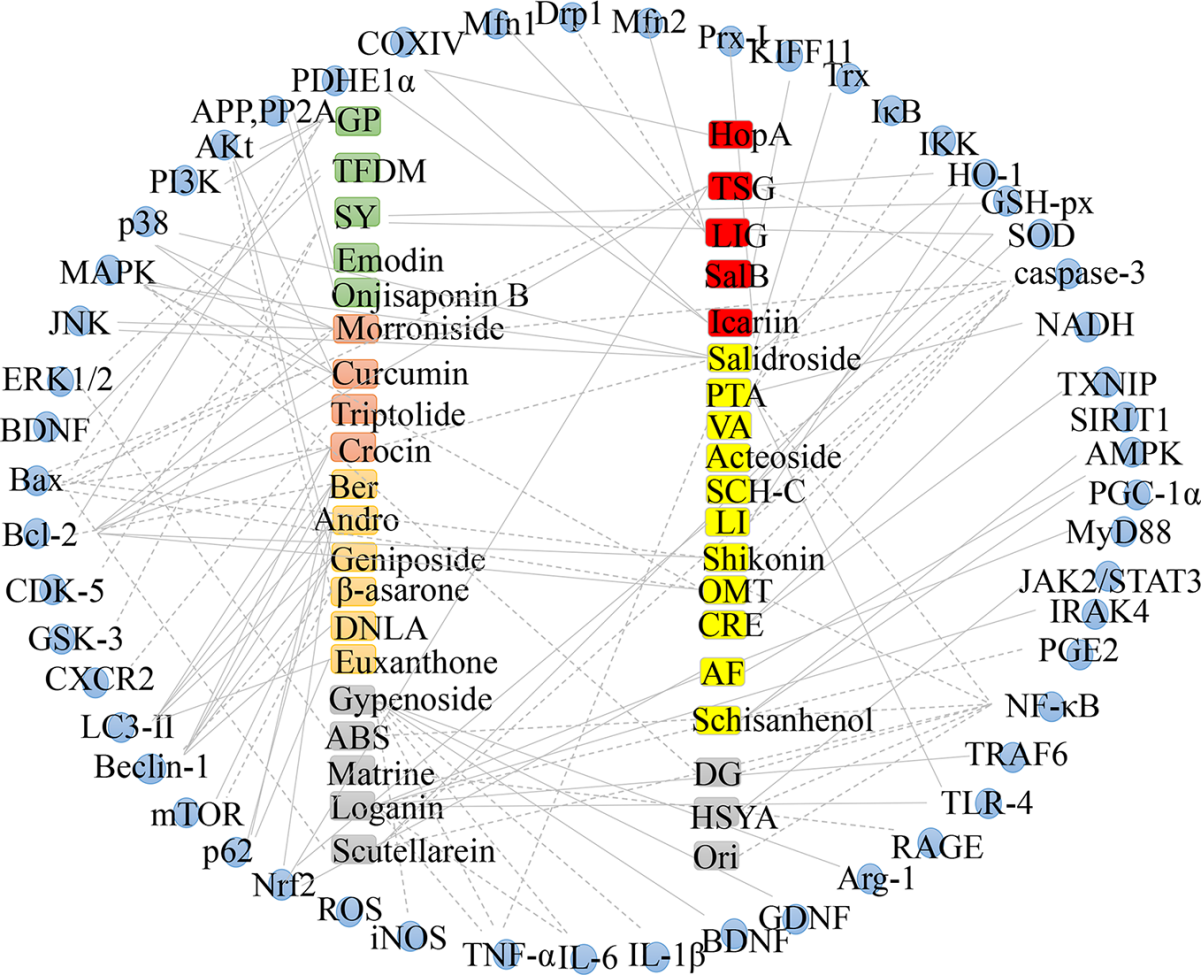
Mechanism in treating AD based on monomer and extract of TCM.

AD is a progressive neurodegenerative disease characterized by progressive loss of memory and cognitive function. Several studies have investigated the pathogenesis of AD in order to develop strategies to treat AD. So far, it has been reported that β-Amyloid, apoptosis, autophagy, neuroinflammation, oxidative stress, and mitochondrial dysfunction participate in the pathogenesis of AD. The current drugs used to control symptoms of AD are often accompanied by side effects and are not sufficiently effective. This is because the of pathogenesis is complicated and is not fully understood. Recent research has provided evidence that natural active ingredients in TCM drugs have multi-target therapeutic effects. Thus, important active monomers and bioactive compounds extracted from TCM herbs have the potential to be new drugs for treating AD. We show that a variety of bioactive components from TCM such as *Polygala tenuifolia*, *Tripterygium wilfordii*, *Andrographis paniculata*, *Gynostemma pentaphyllum*, *Schisandra sphenanthera*, and *Reynoutria multiflora* can improve AD. The monomers and extracts of TCM mentioned in this paper regulate AD by decreasing β-Amyloid production, autophagy, apoptosis, neuroinflammation, oxidative stress as well as mitochondrial dysfunction (**Figure 3**).

In this study, we found that compounds extracted from *Lamiaceae family* are potential treatments for AD. For example, *Dracoephalum moldavica* L., suppresses AD progression by reducing Aβ production, whereas *Scutellaria baicalensis* Georgi. and *Isodon rubescens* (Hemsl.) H.Hara improve AD by preventing neuroinflammation. *Lavandula angustifolia* Mill. and *Rosmarinus officinalis* L. confer therapeutic benefits on AD by reducing oxidative stress whereas *Salvia miltiorrhiza* Bge. suppresses the progression of AD by resolving mitochondrial dysfunction. This may be associated with this plant contains aromatic oils and other active ingredients. In addition, extracts from *Fabaceae* family and *Polygonaceae* family have beneficial effects on AD. It should be noted that TCM exhibit multi-target, multi-pathway, and multi-system characteristics, and their monomers have same mechanism of action but different structures. Among TCM compounds, flavonoids, alkaloids, and polyphenols have the most significant effects on AD. Further studies are advocated to explore further mechanisms of these drugs on AD.

## Author Contributions

S-YC and YG prepared the draft manuscript. J-YS and DY searched the database and extracted literature. L-HF summarized all the tables. LX, PW, and X-LM revised the manuscript. All authors read and approved the manuscript.

## Funding

This work was supported by National Natural Science Foundation of China (NO. 81903902 and 81773974), “Xing Lin scholar” discipline talents, Research ability Enhancement Program of Chengdu University of Traditional Chinese Medicine (BSH2019001) and Project funded by China Postdoctoral Science Foundation (00808412).

## Conflict of Interest

The authors declare that the research was conducted in the absence of any commercial or financial relationships that could be construed as a potential conflict of interest.
